# Correlation of Blood and Salivary pH Levels in Healthy, Gingivitis, and Periodontitis Patients before and after Non-Surgical Periodontal Therapy

**DOI:** 10.3390/diagnostics12010097

**Published:** 2022-01-03

**Authors:** Pradeep Koppolu, Sunkara Sirisha, Soumya Penala, Pathakota Krishnajaneya Reddy, Dalal H. Alotaibi, Ghadah Salim Abusalim, Amara Swapna Lingam, Areej H. Mukhtar, Ali Barakat, Ahmed A. AlMokhatieb

**Affiliations:** 1 Department of Preventive Dental Sciences, College of Dentistry, Dar Al Uloom University, Riyadh 13314, Saudi Arabia; 2 Clove Dental Hospital, Hyderabad 500033, India; sirisha.sunkara@gmail.com; 3 Department of Dentistry, Mahaveer Institute of Medical Sciences, Vikarabad 501101, India; penalasoumya@gmail.com; 4 Department of Periodontics, Sri Sai College of Dental Surgery, Vikarabad 501101, India; drkrishreddy@gmail.com; 5 Department of Periodontics and Community Dentistry, College of Dentistry, King Saud University, Riyadh 11362, Saudi Arabia; dalalotaibi@ksu.edu.sa; 6 Department of Medical Laboratory Science, College of Applied Medical Sciences, Prince Sattam bin Abdulaziz University, Al-Kharj 11942, Saudi Arabia; g.abusalim@psau.edu.sa; 7 Department of Surgical and Diagnostic Sciences, Dar Al Uloom University, Riyadh 13314, Saudi Arabia; lingam@dau.edu.sa; 8 Periodontics Division, Preventive Dentistry Department, Riyadh Elm University, Riyadh 13244, Saudi Arabia; areejmukhtar@hotmail.com; 9 Restorative and Prosthetic Department, College of Dentistry, Dar Al-Uloom University, Riyadh 13314, Saudi Arabia; dr.ali.a.barakat@gmail.com; 10 Conservative Dental Department, College of Dentistry, Prince Sattam bin Abdulaziz University, Al-Kharj 11942, Saudi Arabia; A.almokhatieb@psau.edu.sa

**Keywords:** SRP, saliva, blood, periodontitis, pH, gingivitis

## Abstract

Periodontitis is an infectious illness which leads to the inflammation of protective tissues around the teeth and the continuous loss of alveolar bone and conjunctive tissue. Biomarker analysis in serum and saliva helps in the evaluation of disease progression and activity. It is also established that every inflammatory change along with resultant damage of tissues ends up in altered pH values in the fluids and tissues. Aim: To correlate the connection of pH levels in both blood as well as saliva in healthy, periodontitis, and gingivitis patients. Materials and Methods: The current research involved 145 subjects amidst the age of 20 and 55 years. The subjects were split into three different groups: healthy (Group A), gingivitis (Group B), and finally chronic periodontitis (Group C). The recording of clinical parameters was done by gingival index (GI), probing depth (PD), and plaque index (PI). pH of saliva and blood was analyzed with the help of digital single electrode pH meter. Subjects have gone through scaling and root planning (SRP) coupled with the instructions of oral hygiene. They were recalled post 4 weeks, and saliva and blood samples were gathered for analyzing pH. Results: Clinical parameters GI and PI were statistically important in both group C as well as group B post SRP. A crucial change has been observed in attachment levels (AL) and PD in the case of periodontitis group post SRP. The difference in the salivary pH values were significant between group B vs. C and A vs. C before the treatment because the values for group C were acidic, whereas in groups B and A the pH was alkaline. After the treatment, the values were still significant because the pH has become more alkaline compared to preoperative value in both group B and C. Saliva’s pH levels have demonstrated a statistically significant reduction in group C post SRP. Conclusion: Salivary pH levels and blood evidently became alkaline in the group C patients post SRP and there is a positive correlation between them and the clinical parameters.

## 1. Introduction

Periodontitis is described as an inflammatory illness in the teeth’s supporting tissue which is caused by a group of microorganisms or particular microorganisms, leading to the continuous destruction of periodontal ligament along with alveolar bone with recession, pocket formation, or even both [1]. It seems to be characterized by complicated host-parasite interactions which result in gingival inflammation, periodontal ligament destruction, alveolar bone resorption, and connective tissue attachment. It is the second most familiar oral disorder after dental caries, affecting around 5–30% of the adult population. The history of periodontitis naturally follows an intermittent pattern of remission and exacerbation specified by the activity as well as inactivity of disease [2].

Changes in the environmental and microbial dynamics in terms of microbial ecosystems shall increase the capability of pathogenicity lying inside the microbial ecosystem and later start as well as promote oral disorders. These successional changes have been defined by Marshal in recent times as a hypothesis of ecological plaque. Environmental characteristics decide which metabolic activities and site can be occupied by which microorganism belonging to the microbial communities; this in turn brings changes in the environmental aspects [3].

It is an acknowledged fact that periodontal diseases in humans are principally associated with Gram-negative anaerobic organisms and that, before disparaging periodontal diseases are originated, these microorganisms will colonize tooth surfaces at and apical to the gingival margin. Microbial colonization is a prerequisite for continuous periodontal illnesses. Many studies have observed that periodontal pathogens post colonization tend to grow at particular pH conditions [4].

The diagnosis of active phases in case of periodontal illness along with the recognition of patients who are at threat for active illness illustrate the existing challenge for clinicians as well as clinical analysis. Generally, clinical parameters which include clinical attachment level, probing depth, radiographic loss of alveolar bone, and bleeding on probing plaque index are useful in evaluating the severity of the disease [5].

Closely observing the microbial infection and the assessment of host responses is significant in the identification of people who are at risk of periodontal breakdown. Gingival crevicular fluid, serum, dental plaque, and whole saliva can be utilized as a source of specimens for analyzing the biomarkers which indicate risk [6].

Saliva exerts a greater and vital influence on maturation, metabolism, and plaque initiation. It is a cheap, easy to use, and non-invasive diagnostic technique and its advantages as a diagnostic tool are documented well. The collection of saliva can be done in various forms: a) un-stimulated or resting whole saliva, b) stimulated whole saliva, c) glandular saliva (particularly parotid) with stimulation or without stimulation, sub-lingual/sub-mandibular saliva, and finally d) palatine saliva. Whole saliva comprises secretions produced from the salivary gland coupled with GCF, microorganisms, leukocytes, and desquamated epithelial cells. The secretion of normal whole saliva differs from 800–1500 mL per day or 1.0–3.0 mL per minute, having pH with the range of 6 to 7 in case of un-stimulated whole saliva [7].

The resting pH of the oral cavity is between 5 and 9 and it is also observed to extensively differ based on the number of factors. The pH is uplifted through the loss of carbon dioxide along with ammonia formation via protein degradation and dental plaque bacteria during stagnation. Whilst this circumstance has been researched broadly, and is associated with dental caries, few studies have been carried out on its possible importance with respect to periodontitis and gingivitis.

The diagnosis of dynamic phases of periodontal disease and the identification of patients at jeopardy for active disease signifies a challenge for clinicians. Thus, the goal of the current research is to correlate the connection of saliva’s pH levels and blood in gingivitis, periodontitis, and healthy patients.

## 2. Materials and Methods

The research was carried out in College of Dentistry Institutional ethical committee, which approved this research (COD/IRB/2020/31), approved on 19 December 2020. Using a one-sided type I error of α = 0.025, a sample size of 50 patients in each group ensured a power of 80% for a pairwise group comparison with an effect size of 1.06 standard deviation units in an independent samples *t*-test model. Five subjects were lost to follow up after treatment one from group A and two of them from group B and C. The research population comprised of 145 subjects amidst 22–55-year age category. Subjects with chronic gingivitis, healthy gingiva depending on the clinical appearance, as well as periodontitis having probing depths of 4 mm and more were split into three groups, namely, healthy (Group A), gingivitis (Group B), and finally chronic periodontitis (Group C) comprising 48 subjects in groups B and groups C and 49 subjects in group A.

The nature of the study was orally described to every subject, and an informed written agreement was acquired (according to the Declaration of Helsinki).

Exclusion Criteria:

- Patients who take medication for any sort of medical condition.
- Drugs which involve blood coagulation and any kind of bleeding complications.
- People with a smoking habit.
- Lactating and pregnant women.
- Patients who had gone through oral prophylaxis six months before the research.

The subjects were split into the below mentioned groups:

- Methodically healthy having healthy gingiva: Healthy group (group A) with gingival index (GI) ≤ 0.1
- Chronic gingivitis on the basis of GI ≥ 0.2, without any clinical attachment loss: Gingivitis group (group B)
- Methodically healthy having probing depths of ≥4 mm and clinical attachment loss ≥ 1 mm: group of periodontitis (group C).

Venous blood and un-stimulated saliva from antecubital fossa were gathered prior to the clinical analysis in a sterile container. The estimation of pH was done with the help of digital single electrode pH meter. The clinical analysis comprised GI (Leo and Silness), plaque index (PI) (Silness and Leo), PD, and loss of clinical attachment with complete personal and medical history. Through using UNC 15 probe the PD was measured. All the patients have gone through SRP coupled with the instructions for oral hygiene.

**Figure 1:**
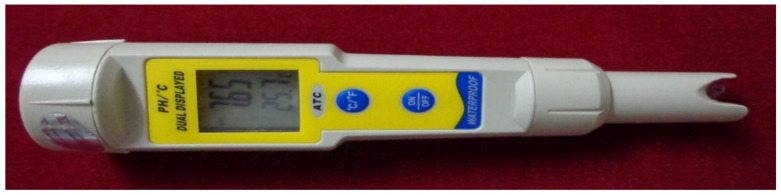
Digital single electrode Ph meter.

The patients were again called post 4 weeks period, and the collection of saliva and blood samples was made for analyzing the pH levels before clinical analysis. The perfect time for collecting saliva and blood for estimation of pH is 1 h, prior to 2 h after consuming food. pH of saliva and blood is analyzed by applying digital single electrode pH meter TECPEL® Taiwan (Figure 1).

### Statistical Analysis

All statistical analyses were performed using IBM SPSS Statistics version 25.0 (IBM Corp. Released 2017. IBM SPSS Statistics for Windows, Version 25.0. Armonk, NY, USA: IBM Corp). The parameters and clinical indices amidst the periodontitis and gingivitis groups were compared through independent sample t test; on the other hand, the pH of blood and saliva among the three groups were compared by assessing the variance having post hoc Games-Howell test. A *p* value of <0.05 was regarded as being statistically significant.

## 3. Results

The research population comprised about 145 people. The evaluation of subjects was done for PD, bleeding, AL and plaque, coupled with salivary and blood pH levels. Table 1, Figure 1, and Table 2 illustrate the summary of the statistics for these sources for every subject.

The pH levels of saliva and blood in healthy group were greatly more alkaline than in periodontitis and gingivitis group. The subjects of periodontitis group demonstrated more acidic pH than gingivitis and healthy group at the baseline. Post SRP, a change in the levels of pH was found in periodontitis as well as gingivitis group that was statistically important for periodontitis group.

After the changes in SRP, pH levels of blood were not statistically significant in group B or group c. Contrary to that, the pH levels of saliva exhibited statistically crucial variation post the periodontitis group of SRP (Table 2).

**Figure 2:**
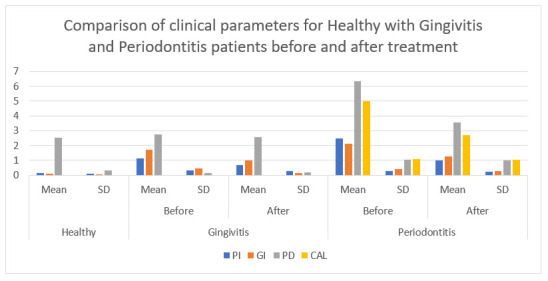
Comparison of parameters for Healthy with Gingivitis and Periodontitis patients before and after treatment.

**Figure 3:**
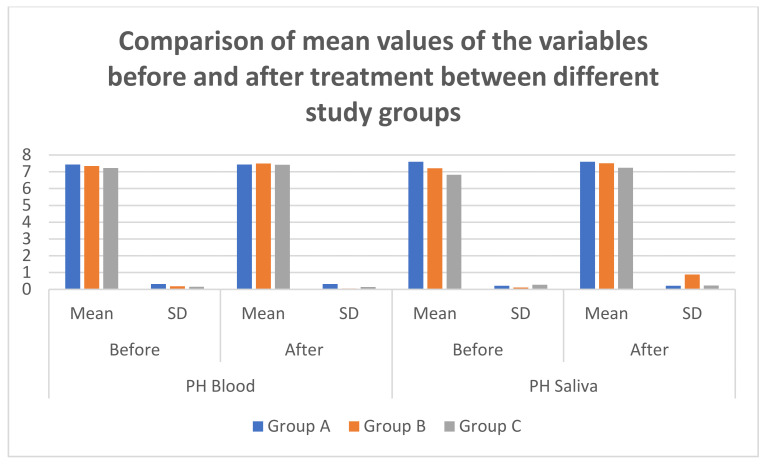
Comparison of mean values of the variables before and after treatment between different study groups.

The mean salivary and blood pH levels seemed to be the least in group C accompanied by the group A and group B at baseline. The mean pH levels of saliva and blood were lesser in group C on making a comparison with group B prior and post SRP (Table 3 and Table 4; Figure 2 and Figure 3). The difference in the salivary pH values was significant between group B vs. C and A vs. C before the treatment as well, because the values for group C were acidic whereas group B and A were alkaline. After the treatment the values were still significant because the pH had become more alkaline compared to preoperative value in groups B and C.

## 4. Discussion

Chronic periodontitis is an infectious as well as inflammatory illness of oral cavity; it is a general periodontal pathology that affects people at various groups of age. Changes in environmental and microbial dynamics in terms of microbial ecosystems increase the capacity of pathogenicity inside the microbial ecosystem and as a result they tend to initiate and promote the oral illness that has been defined by Marsh. It has been considered as the ecological plaque hypothesis [3].

There is a connection between periodontal diseases and gram-negative anaerobic microorganisms for starting the process of disease. With these organisms, gingival margin and tooth surfaces get colonized [8,9]. A study on pH impact on microorganism’s growth analyzed by Takashi et al. [10,11] displayed that the growth of *P. intermedia* occurs at a pH of 5.0–7.0, *F. nucleatum* grows at a pH of 5.5–7.0, and *P. gingivalis* grows at a pH of 6.5–7.0.

Generally, a healthy periodontal and dental condition will be indicated in salivary pH of 7.0, at this specific pH, less incidence of dental decay or else no or little calculus is witnessed. Thus, stable conditions must generally be seen in this environment.

If there is an existence of chronic situation of academia then there is more possibility for the mouth to be highly susceptible to result in dental decay, periodontitis, and halitosis. Salivary pH more than 7.0 generally specifies alkalinity. Over alkalinity can significantly bring in similar kinds of anaerobic situations like academia, yet it seems to be very a rare situation.

In case of subgingival situations, anaerobic and asaccharolytic or/and proteolytic bacteria like *Prevotella*, *Fusobacterium*, *Porphyromonas,* and *Campylobacter* are seen. Nitrogenous compounds can be degraded by proteolytic bacteria into tiny amino acids and peptides through cell-membrane-bound or/and extra-cellularly secreted proteases in order for the ultimate usage as metabolic substrates [12].

The majority of the subgingival microbiota which are responsible for chronic periodontitis either use or form end products. These end products look naturally mild or else averagely acidic: glutamic acid has been utilized by Fusobacterium species as a nutrient and generate butyric and acetic acids; *P. intermedia, C. rectus* and *P. gingivalis* take charge of metabolizing aspartic acid into succinic acid, yet formic acid is needed to be used as a reducing agent [12].

In the current research on salivary and blood pH, it was calculated in gingivitis, periodontitis, and health groups prior to and post SRP. The pH level in blood differed amid various research groups which do not seem to be significant. Our research disclosed the existence of acidic pH in saliva subjects containing periodontitis while having a comparison with healthy group and gingivitis. According to the concept of Takashi et al. [12], the conclusion was stated that periodontal pathogen tends to grow in an averagely acidic environment.

Baliga et al. [13] and Seethalakshmi et al. [14] came up with a conclusion in their studies that the pH level in saliva of patients with chronic periodontitis is observed to be more acidic than the patients with healthy groups and gingivitis. Similar to that, Cormos et al. [15] evaluated gingivitis, healthy and subjects having periodontitis and even witnessed that since the progression of disease on the pH level of saliva acts being acidic natured, denoted the current research analysis.

Contrary to the present research Patel et al. [16] Rajesh et al. [17] ended with a conclusion stating that patients having chronic generalized periodontitis coupled with periodontal pockets are bigger than 4 mm with a moderate pH level in saliva of 11.65 ± 2.26, i.e., greatly alkaline. They had a further suggestion that subjects with salivary mineralization parameters increase such as phosphorous and salivary calcium, greater pH level in the saliva are possibly at greater risk of getting periodontitis.

pH levels of saliva and blood were calculated post SRP in the patients with periodontitis and gingivitis. SRP ended up in the total increase of pH in blood and saliva and it was observed to be statistically crucial in terms of periodontitis group. After the treatment, comparison of these three groups has disclosed the crucial variation in the values of SRP pH. The outcomes have recommended that pH of saliva return back to neutral or alkaline levels after the treatment, in correspondence with the changes in gingival, plaque, and periodontal parameters.

Gracia et al. [18] in a study reviewed an alkaline pH present in the patients with gingivitis and periodontitis and acidic or neural pH in healthy set of people. In addition to that they gave the conclusion that, after the therapy, a reduction in salivary pH was observed.

Govindraj et al. [19] assessed viscosity and salivary pH differences between working and nonworking men and women and evaluated the effect of stress on salivary pH and viscosity. They have concluded that stress exerts its influence on salivary pH, flow rate, and viscosity.

A study of Orozco et al., made in recent times [20], came up with the conclusion that salivary pH patients with periodontitis are somewhat alkaline, and differ after the periodontal treatment which tends to get it restored to the normal levels, responding to the periodontal tissue repair. Similarly, the pH differs in maintaining a direct association with certain periodontal pockets and biofilm index.

A more recent research salivary flow rate and pH of periodontal patients with associated cardiovascular disease concluded that decrease in salivary pH and flow rate was significantly associated with severity of periodontal disease [21]. The present study protocol can also be used in patients getting the rehabilitation with implants as well [22,23,24,25,26,27]. Titanium creates an extremely shielding oxide film on the surface, and it becomes passive. Therefore, titanium shows exceptional corrosion resistance in artificial saliva. Since the contemporary research area is focusing on the presence of corrosive titanium products approximating peri-implant tissues as it could be a potential risk factor for peri-implantitis, the present study can be applied for in vitro or in vivo studies related to implants as well.

The limitations of the present study include small sample size. Subjects were reevaluated 4 weeks after treatment; long term surveillance after treatment would give further insights into salivary and blood pH levels.

## 5. Conclusions

Blood pH levels differences were not significantly different between the study groups, whereas salivary pH levels were significantly decreased in terms of periodontitis group post SRP and had a positive correlation with that of the clinical parameters. The difference in the salivary pH values was significant between group B vs. C and A vs. C before the treatment. The values for group C were acidic whereas in group B and A pH was alkaline. After the treatment the values were still significant because the pH became more alkaline compared to preoperative value in group B and C. Thus, the present research demonstrates how useful is the salivary pH as a diagnostic tool for examining periodontal health as well as disease.

Further studies in periodontitis and gingivitis patients after different treatment modalities including SRP in conjunction with antimicrobial and antibiotic therapy and surgical therapy gives us further insights into the effect of therapy on pH levels of saliva and blood.

## Figures and Tables

**Table 1:** Comparison of parameters for healthy with gingivitis and periodontitis patients before and after treatment.

Parameter	Healthy	Gingivitis Mean ± SD		*p*-Value	Periodontitis Mean ± SD		*p*-Value
		Before SRP	After SRP		Before SRP	After SRP	
**PI**	0.13 ± 0.11	1.15 ± 0.34	0.67 ± 0.30	0.036 *	2.48 ± 0.27	0.98 ± 0.25	0.039 *
**GI**	0.09 ± 0.07	1.72 ± 0.46	0.98 ± 0.15	<0.001 ^#^	2.10 ± 0.41	1.26 ± 0.26	<0.001 ^#^
**PD**	2.53 ± 0.34	2.74 ± 0.13	2.56 ± 0.20	0.031 *	6.36 ± 1.03	3.55 ± 1.02	0.027 *
**CAL**		-----	------	------	4.98 ± 1.07	2.70 ± 1.04	0.043 *

#  *p* ≤ 0.001 highly significant; * *p* ≤ 0.05 statistically significant.

**Table 2:** Differences between pH in saliva and blood of healthy, before and after periodontal treatment in gingivitis and periodontitis patients.

Parameter	Healthy Mean ± SD	Gingivitis Mean ± SD		*p*-Value	Periodontitis Mean ± SD		*p*-Value
		Before SRP	After SRP		Before SRP	After SRP	
**pH blood**	7.44 ± 0.32	7.35 ± 0.19	7.49 ± 0.06	0.98	7.22 ± 0.16	7.42 ± 0.14	0.12
**pH saliva**	7.6 ± 0.21	7.21 ± 0.11	7.51 ± 0.88	0.35	6.82 ± 0.27	7.24 ± 0.23	<0.001 *

* *p* ≤ 0.05 statistically significant.

**Table 3:** Comparison of mean values of the variables (before treatment) between different study groups.

	GROUP	Mean ± SD	*p*-Value	Significant Groups at 5% Level INTERVAL
**pH Blood**	A	7.44 ± 0.32	1.09	A vs. B
	B	7.35 ± 0.19	0.12	B vs. C
	C	7.22 ± 0.16	0.04 *	A vs. C
**pH SALIVA**	A	7.6 ± 0.21	0.37	A vs. B
	B	7.21 ± 0.11	0.005 *	B vs. C
	C	6.82 ± 0.27	0.004 *	A vs. C

* *p* ≤ 0.05 statistically significant.

**Table 4:** Comparison of mean values of the variables (after treatment) between different study groups.

	GROUP	Mean ± SD	*p*-Value	Significant Groups at 5% Level Interval
**pH Blood**	A	7.44 ± 0.32	0.52	A vs. B
	B	7.49 ± 0.06	0.34	B vs. C
	C	7.42 ± 0.14	0.15	A vs. C
**pH SALIVA**	A	7.6 ± 0.21	0.78	A vs. B
	B	7.51 ± 0.88	0.004 *	B vs. C
	C	7.24 ± 0.23	0.007 *	A vs. C

* *p* ≤ 0.05 statistically significant.

## Data Availability

The data presented in this study are available upon request.
